# Quantitative Proteomic Analysis of Oral Brush Biopsies Identifies Secretory Leukocyte Protease Inhibitor as a Promising, Mechanism-Based Oral Cancer Biomarker

**DOI:** 10.1371/journal.pone.0095389

**Published:** 2014-04-18

**Authors:** Ya Yang, Nelson L. Rhodus, Frank G. Ondrey, Beverly R. K. Wuertz, Xiaobing Chen, Yaqin Zhu, Timothy J. Griffin

**Affiliations:** 1 Department of General Dentistry, Ninth People’s Hospital, School of Stomatology, Shanghai Jiao Tong University School of Medicine, Shanghai Key Laboratory of Stomatology, Shanghai, China; 2 Oral Medicine, Diagnosis and Radiology, School of Dentistry, University of Minnesota, Minneapolis, Minnesota, United States of America; 3 Department of Otolaryngology, Medical School, University of Minnesota, Minneapolis, Minnesota, United States of America; 4 Department of Oral Medicine, Guanghua School of Stomatology, Sun Yat-sen University, Guangzhou, China; 5 Department of Biochemistry, Molecular Biology, and Biophysics, University of Minnesota, Minneapolis, Minnesota, United States of America; Moffitt Cancer Center, United States of America

## Abstract

A decrease in the almost fifty percent mortality rate from oral cancer is needed urgently. Improvements in early diagnosis and more effective preventive treatments could affect such a decrease. Towards this end, we undertook for the first time an in-depth mass spectrometry-based quantitative shotgun proteomics study of non-invasively collected oral brush biopsies. Proteins isolated from brush biopsies from healthy normal tissue, oral premalignant lesion tissue (OPMLs), oral squamous cell carcinoma (OSCC) and matched control tissue were compared. In replicated proteomic datasets, the secretory leukocyte protease inhibitor (SLPI) protein stood out based on its decrease in abundance in both OPML and OSCC lesion tissues compared to healthy normal tissue. Western blotting in additional brushed biopsy samples confirmed a trend of gradual decreasing SLPI abundance between healthy normal and OPML tissue, with a larger decrease in OSCC lesion tissue. A similar SLPI decrease was observed *in-vitro* comparing model OPML and OSCC cell lines. In addition, exfoliated oral cells in patients’ whole saliva showed a loss of SLPI correlated with oral cancer progression. These results, combined with proteomics data indicating a decrease in SLPI in matched healthy control tissue from OSCC patients compared to tissue from healthy normal tissue, suggested a systemic decrease of SLPI in oral cells correlated with oral cancer development. Finally, *in-vitro* experiments showed that treatment with SLPI significantly decreased NF-kB activity in an OPML cell line. The findings indicate anti-inflammatory activity in OPML, supporting a mechanistic role of SLPI in OSCC progression and suggesting its potential for preventative treatment of at-risk oral lesions. Collectively, our results show for the first time the potential for SLPI as a mechanism-based, non-invasive biomarker of oral cancer progression with potential in preventive treatment.

## Introduction

Unfortunately, the survival rate for people diagnosed with oral cancer, predominantly in the form of oral squamous cell carcinoma (OSCC), is only slightly better than 50%[1]. OSCC is preceded by the occurrence of an oral premalignant lesion, commonly leukoplakia, which transforms to invasive cancer in 5% to 17% of the cases[2], [3]. If diagnosed early, preventive treatments are more effective, increasing the survival rate to 80% or better[4]. Thus, there is a pressing need for better ways to diagnose and treat at-risk OPML and/or early-stage OSCC oral lesions[2].

Invasive incisional biopsy followed by histopathology is the current gold standard for oral cancer diagnosis[5]. Unfortunately, it has numerous limitations. The invasive and costly nature leads to less frequent testing of suspicious lesions, and consequently, a delayed diagnosis of OSCC[6], [7]. One retrospective study found only about a 14% follow-up rate for scalpel biopsies within a 3 year period[2]. Additionally, scalpel biopsy is prone to under-sampling of lesions[8], [9], thereby leading to errors in diagnosis.

Given these limitations of scalpel biopsy, much attention has been given to identifying molecular biomarkers indicative of disease in non-invasively collected patient samples[10]. One promising non-invasive sampling method is the use of brush biopsies[11], [12]. Here, a relatively stiff brush is used to gently collect a sample of trans-epithelial cells directly from the oral lesion, or matched oral mucosa. This collection is simple and cheap, with minimal discomfort to the patient. Most importantly it provides a potentially information-rich sampling of cells directly from the lesion which can be further analyzed[11], [12].

To develop non-invasively collected molecular biomarkers from brush biopsies, promising candidate molecules within these samples must first be identified. Large-scale technologies for molecular profiling (e.g. genomics, proteomics) can identify such candidates. In particular, analysis using mass spectrometry-based proteomics could provide not only leads on actionable protein biomarkers from these samples, but also underlying knowledge of cancer progression mechanisms and possible targets for treatment. However, the proteomic analysis of oral brush biopsies via MS-based proteomics has seen limited attention[13], [14], especially using the most contemporary technologies in the field. To date, no one has applied quantitative shotgun MS-based proteomics, arguably the most versatile and in-depth method for characterizing proteomes[15], to oral brush biopsy analysis.

In this study, we have applied quantitative shotgun MS-based proteomics to the analysis of brush biopsies collected from healthy normal tissues, OPML, and OSCC. Among a number of replicated proteins showing abundance differences, the secretory leukocyte protease inhibitor (SLPI) protein showed dramatic decrease relative to normal tissues correlated with the steps of oral cancer progression. This decreased abundance of SLPI was verified via western blotting in brush biopsy samples, and was also observed in exfoliated cells in whole saliva from OPML and OSCC patients. Consistent with patient results, model cell lines of OPML and OSCC also showed a decrease in SLPI. Additionally, treating a model OPML cell line with SLPI showed an inhibition of NF-κB activity, a transcription factor known to play a role in inflammatory mechanisms underlying oral cancer development. Collectively, our results show for the first time a progressive loss of SLPI abundance in the transition from OPML to OSCC, and suggest a novel role for SLPI as a mechanism-linked, non-invasive biomarker of oral cancer, with potential as an OPML treatment agent.

## Materials and Methods

### Patients and Specimens

The study was done with informed written consent of all sample donors using a human subject protocol approved by the Institutional Review Board at the University of Minnesota (IRB study number 0001M34501). All saliva collections were done without stimulation via passive drooling, during the day between the hours of 9∶00 am and 5∶00 pm. Whole saliva and brush biopsies were collected from 11 patients diagnosed with a dysplastic OPML and 11 patients with OSCC at the University of Minnesota Otolaryngology clinic. For each patient, saliva samples were first collected, followed by collection of the brush biopsies from the lesion and the healthy mucosa of corresponding contralateral area, using Rovers Orcellex brushes (Rovers Medical Devices B.V., Netherlands). Brush biopsies from oral mucosa, and whole saliva were also collected from 10 healthy volunteers. The healthy volunteers had no major risk factors for OSCC (non-smokers, moderate to low alcohol use) and were free of oral lesions. Immediately after collection, samples were stored at −20°C, and then transferred to −70°C until use. Furthermore, information on tobacco and alcohol use of the patients was collected. The characteristics of the study population are summarized in **Table S1**.

### Cell Lines

MSK Leuk1 cells[16], grown from buccal mucosa adjacent to oral leukoplakia lesions, were a gift from Dr. Peter Sacks, New York University. MSK Leuk1 cells were grown in KGM-2 Medium (Lonza Walkersville, MD) supplemented with bovine pituitary extract, recombinant human epidermal growth factor, recombinant human insulin, hydrocortisone, epinephrine, and transferrin at 37°C in 5% CO_2_.

Transformed human epidermal keratinocytes (Rhek) immortalized by Ad 12-SV40 virus[17] were obtained from Dr. Jhong S. Rhim at the National Cancer Institute, (Frederick, MD). The OSCC cell line CA-9-22[18], was a gift from Toshio Kuroki, MD. Cells were maintained in Eagle’s minimal essential medium supplemented with 10% fetal bovine serum, L-glutamine (5.8 mg/ml), and penicillin/streptomycin (50 µg/ml) at 37°C in 5% CO_2_ as adherent monolayer cultures.

### Brushed Biopsy Sample Preparation

**Figure 1:**
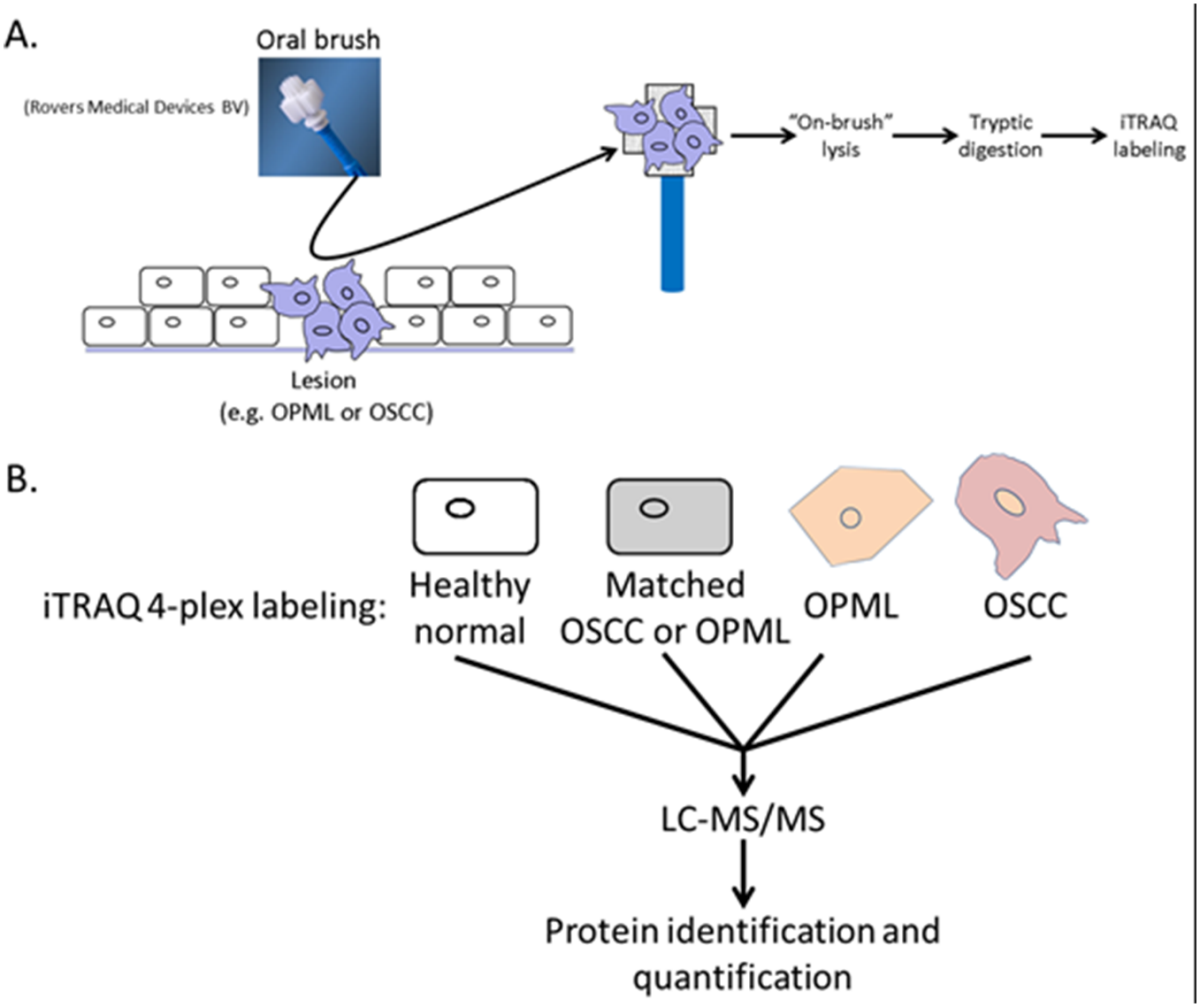
A. Brush biopsy collection and sample preparation protocol. B. Experimental design for quantitative MS-based proteomics experiments. One experiment used matched tissue from OPML patients, while the second experiment used matched tissue from OSCC patients.

For MS-based proteomic discovery studies and western blot validation studies, brush biopsy samples from subjects falling in the two groups were prepared identically. In order to maximize recovery of peptides and minimize sample handling steps, we used an “on-brush” digestion method to produce a peptide solution for MS-based proteomics analysis (see Figure 1A in Results section). The brush head was submerged and lysed in 50 mM tris pH 8.0 with 2% SDS at 95°C for 10 min with intermittent vortexing. Cellular debris was removed by centrifuging at 16 100×g and recovering the supernatant into a clean microfuge tube. Protein recovery was measured using the BCA assay (Thermo Scientific). The recovered proteins were then digested and processed for subsequent mass spectrometry analysis or used for western blot validation.

### Isobaric Peptide Tagging and Sample Preparation

Proteins from brushed cells were reduced in DTT for 1 h at 55°C and trypsin digested using a modified FASP protocol[19]. Iodoacetamide was used as the cysteine alkylating reagent. The resulting peptides were desalted using solid-phase extraction cartridges (tC18 Sep-pak, Waters). Peptides were then dissolved in the manufacturer-supplied buffer and labeled with iTRAQ reagent (Applied Biosystems) at room temperature for 1 h and desalted with Sep-Pak cartridges.

Samples were next fractionated by off-line semipreparative HPLC. The combined, iTRAQ-labeled peptides were resuspended in Buffer A (10 mM ammonium formate pH 10 in 98∶2 water:acetonitrile) and fractionated offline by high pH C18 reversed-phase (RP) chromatography[20]. A MAGIC 2002 HPLC (Michrom BioResources, Inc., Auburn, CA) was used with a C18 Gemini-NX column, 150 mm×2 mm internal diameter, 5 um particle, 110 Å pore size (Phenomenex, Torrence, CA). Buffer A was 10 mM ammonium formate, pH 10 in 98∶2 water:acetonitrile and Buffer B was 10 mM ammonium formate, pH 10 in 10∶90 water:acetonitrile. The flow rate was 150 µl/min. with a gradient from 0–35% Buffer B over 60 min., followed by 35–60% over 5 min. Fractions were collected every 2 minutes and UV absorbance was monitored at 215 nm and 280 nm. Peptide containing fractions were divided into two equal numbered groups, “early” and “late”. The first “early” fraction was concatenated with the first “late” fraction, and so on. Concatenated samples were dried in vacuo, resuspended in load solvent (98∶2∶0.01, water:acetonitrile:formic acid) prior to mass spectrometric analysis.

### Mass Spectrometric Analysis

A linear ion trap-Orbitrap (LTQ-Orbitrap) Velos instrument (Thermo Fisher Scientific)[21] was used for mass spectrometry. The instrument was operated in a top-ten data dependent mode employing survey scans at 30,000 resolution from 300 to 1800 m/z. Tandem MS (MS/MS) scans were acquired with an isolation width of 2 m/z and higher energy collisional dissociation (HCD) fragmentation mode. 40% normalized collision energy was used with a 20 millisecond duration. The automatic gain control settings were 3×10^5^ ions in the ion trap, and 1×10^6^ in the Orbitrap. Dynamic exclusion was used with duration of 15 seconds and a repeat count of 1.

### Protein Identification and Quantification

Raw files were converted to mzXml using msconvert (distributed as part of ProteoWizard 1.6.1260). MS/MS spectra were searched against the Uniprot human database including scrambled sequences and common contaminant proteins (a total of 136,002 entries) using Sequest v27.0. Search parameters included a 1.6 amu (atomic mass units) precursor and 0.8 amu fragment mass tolerance, 2 missed cleavages, partial trypsin specificity, fixed modifications of carbamidomethylated cysteine, iTRAQ reagent modification at lysines and N-termini, and variable modification of methionine oxidation. Search results were filtered to 99% protein probability and 95% peptide probability in Scaffold (v3.3.1, Proteome Software), producing a false discovery rate of 1%. Proteins were quantified using customized software developed in-house[22]. Only proteins identified from two or more MS/MS spectra matched to peptides were considered for quantitative analysis. P-values were assigned to each protein quantified by three or more MS/MS spectra, as described[22]. **Table S2** shows all information for proteins identified and quantified in these experiments.

### Western Blotting Experiments

For western blotting experiments, an independent set of samples was used due to lack of sample material from the initial sample set used in MS-based proteomic experiments. Thirty micrograms (ug) of brush biopsy protein from each individual subject analyzed in validation experiments, or fifty ug of protein from cell lysates of cell lines, along with thirty ug of positive control protein, were separated by 12% SDS-PAGE. Proteins were then transferred to a PVDF membrane (Millipore), and probed with polyclonal rabbit anti-SLPI antibody (1∶250; Abcam ab46763). The blots were labeled with horseradish peroxidase-conjugated secondary antibodies (1∶10,000) and visualized with an ECL detection system (Thermo Scientific).

In the case of whole saliva, unstimulated samples were collected from an independent set of subjects (4 healthy volunteers, 5 patients with OPML and 5 patients with primary OSCC). Whole saliva was centrifuged at 3000×g at 4°C, the supernatant containing the soluble fraction of saliva proteins was collected, and the cell pellets washed with PBS and lysed to obtain cellular proteins. Total protein was quantified using the BCA assay (Thermo Pierce).

### Reporter Gene Assays

The cell lines were plated at 50,000 cells/well in 12 well plates and transiently co-transfected via TransIT Express Reagent (MirusBio, Madison, WI) with a pIgκB-Luc reporter gene plasmid 24 hours later along with a pCMV Lac-Z reporter containing the CMV promoter and Lac-Z gene in pcDNA3 to adjust for transfection efficiency. The pIgκB-Luc reporter construct contains three immunoglobulin G-κ chain NF-κB binding sites driving the luciferase gene and was kindly provided to us by Dr. K. Brown (NIAID, NIH). After overnight transfection, cells were treated with recombinant human SLPI (R&D systems, Minneapolis, MN). Cell lysates were analyzed via Tropix Dual Light Reporter Gene Assay (Applied Biosystems, Carlsbad, CA) on a Tristar dual injection flash luminometer (Berthold Technologies, Oak Ridge, TN). Nine replicates were measured per data point.

## Results

### Profiling Oral Cancer Progression-associated Protein Dynamics via MS-based Quantitative Proteomics

Two rounds of quantitative MS-based proteomics were employed (**Figure 1B**), using isobaric peptide labeling with the iTRAQ reagent[23] to analyze soluble proteins isolated from whole cell lysates from oral brush biopsy samples. The first compared separate protein mixtures pooled from two OPML tissues, two matched OPML control tissues, two OSCC tissues and two healthy normal tissues. The second compared separate protein mixtures pooled from four OPML tissues, four OSCC tissues, four matched OSCC control tissues and four healthy normal tissues. The sample design was determined primarily by availability of clinical samples, the amount of protein available from each brush biopsy and our goal to compare all different types of tissues available. A total of 643 and 1164 proteins were identified and quantified, for the first and second iTRAQ analysis and respectively. The increased number of proteins identified in the second analysis was most likely due to increased total protein due to pooling of more patient samples compared to the first analysis. **Table S2** shows information on all proteins identified and quantified in these experiments.

To prioritize proteins for subsequent validation experiments, we looked at abundance changes for OPML or OSCC tissues compared to the healthy normal control tissues in each iTRAQ experiment. We further constrained these results by looking for only those proteins that showed consistent relative abundance differences in both iTRAQ experiments. A total of 21 and 15 proteins met these criteria for OPML and OSCC tissues, respectively (**Table** **1**). Despite the recognized phenomena of abundance ratio compression due to precursor interference in isobaric peptide tagging-based studies[24], [25], we observed a number of rather large relative abundance changes (>2-fold) in our study. Interestingly, only three of these proteins showed abundance changes in both tissue types (OPML and OSCC) compared to healthy normal (bold text in **Table 1**).

**Table 1:** Differentially abundant proteins in OPML and OSCC lesion tissues compared to healthy control tissues.

Symbol	Protein name	OPML 2*	OPML 1*	OSCC 2*	OSCC 1*
ATP5A1	ATP synthase, H+ transporting, mitochondrial F1 complex, alpha subunit 1, cardiac muscle	1.506	1.575	−	−
**CSTA**	**cystatin A (stefin A)**	**3.166**	**1.966**	**−1.525**	**−9.198**
IGHA2	immunoglobulin heavy constant alpha 2 (A2m marker)	1.967	2.180	−	−
IGJ	immunoglobulin J polypeptide, linker protein for immunoglobulin alpha and mu polypeptides	2.572	1.861	−	−
JUP	junction plakoglobin	−1.569	−2.825	−	−
KRT3	keratin 3	−2.415	−7.325	−	−
KRT14	keratin 14	−2.131	−3.223	−	−
KRT16	keratin 16	−2.149	−2.501	−	−
**KRT36**	**keratin 36**	**−1.780**	**−4.255**	**−1.896**	**−16.155**
KRT76	keratin 76	−2.620	−5.663	−	−
KRT80	keratin 80	−1.519	−4.345	−	−
KRT84	keratin 84	−2.085	−6.578	−	−
KRT6A	keratin 6A	−1.501	−3.890	−	−
POF1B	premature ovarian failure, 1B	−2.137	−3.060	−	−
PPIA	peptidylprolyl isomerase A (cyclophilin A)	−1.517	−1.800	−	−
PRB1/PRB3	proline-rich protein BstNI subfamily 1	2.043	1.782	−	−
RPL15	ribosomal protein L15	−1.572	−1.989	−	−
RPTN	repetin	−1.880	−7.139	−	−
**SLPI**	**secretory leukocyte peptidase inhibitor**	**−33.717**	**−5.259**	**−34.577**	**−14.813**
SMR3B	submaxillary gland androgen regulated protein 3B	3.180	3.215	−	−
WDR1	WD repeat domain 1	1.734	2.579	−	−
ANXA1	annexin A1	−	−	−1.575	−3.171
ANXA2	annexin A2	−	−	−1.570	−2.671
EPS8L1	EPS8-like 1	−	−	−1.704	−8.073
FLG	filaggrin	−	−	−1.575	−12.127
HBB	hemoglobin, beta	−	−	1.689	2.428
IL36A	interleukin 36, alpha	−	−	−1.703	−31.421
KRT9	keratin 9	−	−	−1.511	−15.191
RPS23	ribosomal protein S23	−	−	1.557	1.789
SERPINA1	serpin peptidase inhibitor, clade A (alpha-1 antiproteinase, antitrypsin), member 1	−	−	1.628	2.638
SPINK5	serine peptidase inhibitor, Kazal type 5	−	−	−1.579	−12.235
SPRR1B	small proline-rich protein 1B	−	−	−1.719	−19.939
TRIM29	tripartite motif containing 29	−	−	1.509	1.629

*fold-change for each OPML or OSCC tissue replicate compared to healthy normal tissue.

**Figure 2:**
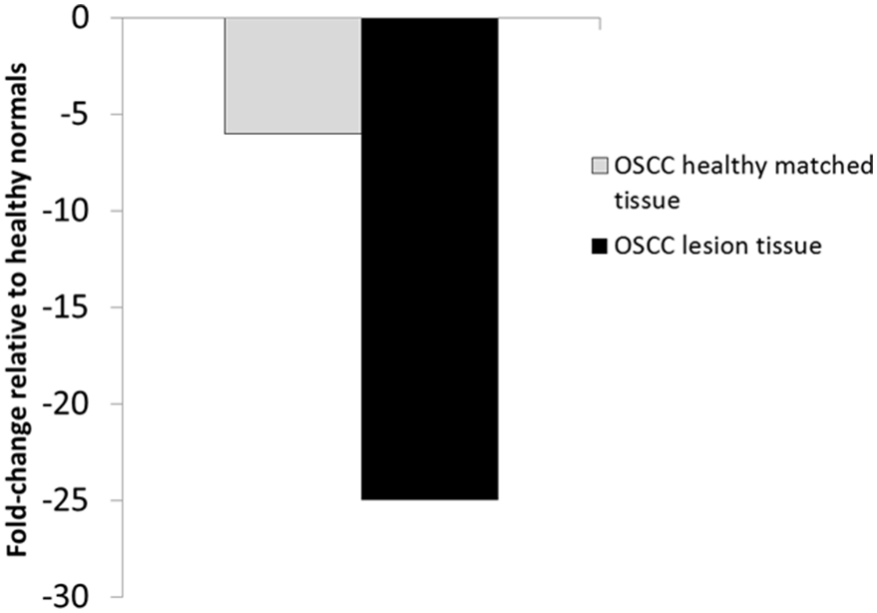
Average SLPI relative abundance levels relative to healthy normal tissue in OSCC matched healthy tissue and OSCC lesion tissue.

Of those proteins shown in Table 1, the Secretory Leukocyte Protease Inhibitor (SLPI), stood out based on its large decrease in both OPML and OSCC tissues when compared to healthy normals (19.5 and 12.4 average abundance decrease for OPML and OSCC tissues, respectively). Based on these findings, and the known role of SLPI as a protease inhibitor with connections to oral cancer[26], we chose to further validate and investigate this protein. To this end we first further interrogated the quantitative proteomics data on SLPI. Of note were the results from the second iTRAQ experiment which included the matched OSCC healthy control tissue. The results showed that not only was SLPI decreased in the OSCC lesion tissue compared to healthy normals, but also it was decreased in the matched healthy tissue compared to the healthy normal tissues (**Figure 2**). A relatively small decrease was also observed in the first iTRAQ experiment between matched tissue from OPML patients and healthy normal tissues (**Table S2**).

### Validating Cancer Progression-dependent SLPI Abundance Dynamics in Independent Samples

**Figure 3:**
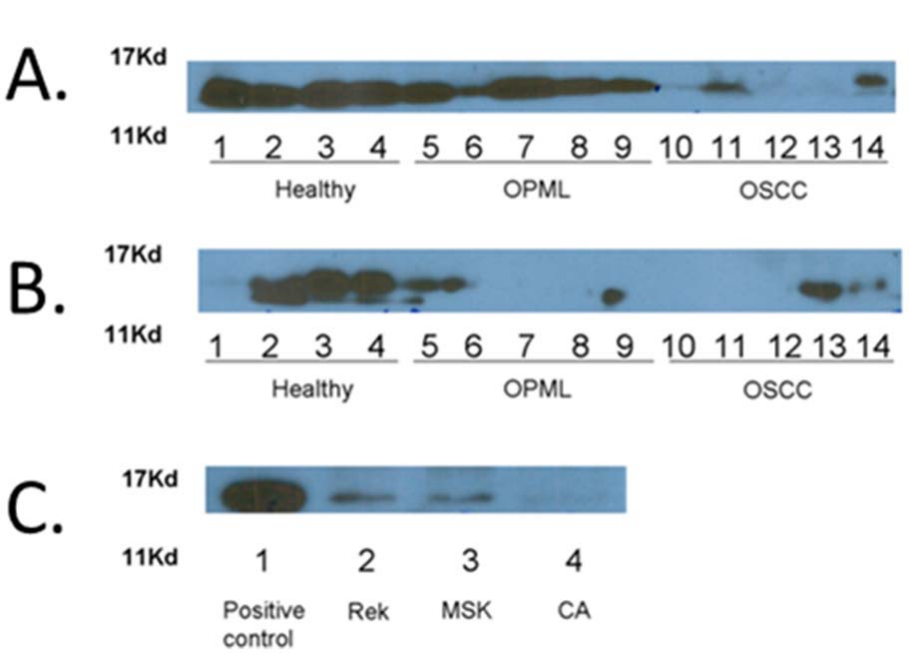
Western blotting results against SLPI. A. Verification of decreased SLPI in tissues collected by brush biopsy. B. SLPI abundance levels measured in exfoliated cells from whole saliva. C. SLPI abundance levels measured in model cell lines. Positive control is a normal healthy human saliva sample; Rhek cells are from a normal epithelial cell line, MSK is a model OPML cell line (MSK-Leuk1) and CA9-22 is a model OSCC cell line.

In order to validate the observed abundance decrease of SLPI in both OPML and OSCC tissues, we first pursued semi-quantitative western blotting experiments in additional brush biopsy samples. As shown in **Figure 3A**, the western blots confirmed the MS-based results, as SLPI abundance showed a gradual decrease between healthy normal tissue and OPML tissue, with a more dramatic decrease in OSCC tissues. **Figure S1** shows loading control results via total protein staining of membranes used for results shown in Figure 3. Additionally, we collected un-stimulated whole saliva samples from the same patients who consented to brush biopsies. We isolated the exfoliated cells in these samples via centrifugation and probed the isolated proteins for SLPI after cell lysis. The results showed a similar trend in abundance decrease of SLPI between healthy normal tissues and both OPML and OSCC tissues (**Figure 3B**). Analysis of the soluble proteins contained in saliva supernatants showed a less consistent trend (data not shown).

We also tested abundance of SLPI protein in model OPML and OSCC cell lines (**Figure 3C**). Here, we chose to compare soluble proteins isolated from whole cell lysates from control RHEK cells, MSK-leuk1 cells (a model cell line of OPML[27]) and Ca9-22 cells (a model cell line of OSCC[28]). Similar to the results from the patient brush biopsies, we observed a dramatic decrease in SLPI in the MSK-leuk1 and Ca9-22 cell lines compared to healthy control cells.

### Testing Potential Anti-inflammatory Effects of SLPI Treatment on OPMLs

**Figure 4:**
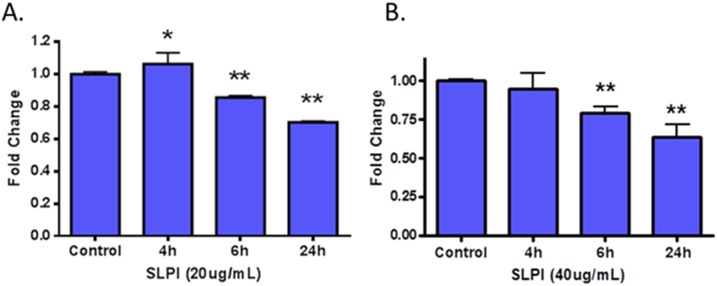
Effect of SLPI peptide treatment on NF-κB activity in an MSK-Leuk1 cell line at two different doses. Fold-change of an NF-κB reporter gene assay relative to vehicle control is plotted at different time points after treatment.

The role of NF-κB activation and regulation of pro-inflammatory factors in the mechanism underlying transition from OPML to OSCC is well-known[29], [30], [31]. Evidence exists showing that SLPI inhibits NF-κB[32], [33], although this has not been demonstrated in models of oral cancer. Given this evidence, we sought to investigate whether or not SLPI decreases NF-κB activity in MSK-Leuk1 cells, using a luciferase-based reporter assay[34]. We treated the transfected MSK Leuk1 cells with two different concentrations of pure SLPI peptide (20 ug/mL and 40 ug/mL), and measured NF-κB at different times after treatment (**Figure 4**). Results for both treatments were comparable, with both showing approximately a 40% drop in NF-κB after 24 hours of SLPI treatment. Treatment with a lower amount of SLPI (10 ug/mL) exhibited a smaller and less reliable decrease in NF-κB activity (data not shown).

## Discussion

We have conducted a first-of-its-kind, in-depth quantitative shotgun proteomics analysis of non-invasively collected oral brush biopsy samples, seeking to identify protein abundance changes associated with oral cancer progression. Brush biopsies are well-known for their value as a non-invasively collected cellular sample for oral cancer diagnostic applications[11], [12]. However, MS-based proteomic studies taking advantage of these potentially information-rich samples have been limited. Notably, Driemel et al[13] used surface-enhanced laser desorption/ionization (SELDI) MS to quantitatively profile brush biopsy samples and identify potential biomarkers of progression. Remmerbach et al[14] used more standard MALDI-MS to analyze proteins isolated from brush biopsies as well. Although these studies had some success, the use of a SELDI or MALDI-based methods on complex mixture is limited to a subset of relatively small proteins and/or peptides in the samples of interest.

MS-based shotgun proteomics on the other hand offers an expanded view of brush biopsy proteomes, given its ability to identify and quantify proteins of all molecular weight classes[15]. Our study provides the first demonstration of shotgun proteomics applied to oral brush biopsy samples. One question at the outset of this study was whether cells collected via brush biopsy would provide ample total protein for a large-scale proteomic analysis. We found that an on-brush cell lysis method provided better yield (at least tens of micrograms of total protein) than attempting to wash cells free of the brush prior to lysis (data not shown). Our findings should open the way for additional shotgun proteomic analyses of brush biopsies for characterizing oral lesions in different contexts.

Our replicate analyses comparing healthy normal tissue to OPML and OSCC tissue, as well as matched controls, revealed a number of interesting proteins showing changes in abundance. Although other candidates worthy of further validation were identified, we chose to focus on SLPI, given its large and reproducible decrease in abundance in both OPML and OSCC tissues when compared to healthy normal tissue. Researchers have traditionally acknowledged the role of SLPI in inhibiting serine proteases; however, knowledge of its functions have continued to expand to include antimicrobial, immunity and anti-inflammation roles[35]. SLPI’s role in oral cancer progression is less known, however, recent results in biopsied tissue slices demonstrated a decrease in SLPI in OSCC tissues compared to healthy, as well as a potential role in inhibiting invasiveness[26].

Our study has revealed several new findings about SLPI’s possible role in oral cancer. For one, our results are the first to demonstrate a significant drop in SLPI in non-invasively collected oral brush biopsy samples, as well as the cellular fraction of whole saliva. These findings are consistent with recent results showing a SLPI abundance decrease in OSCC tissue slices collected via traditional scalpel biopsy[26]. Importantly our study is the first to examine both OPML and OSCC tissues, demonstrating a progressive loss of SLPI in the pre-malignant state, followed by a more dramatic decrease OSCC. Our results suggest a potential role for SLPI as a non-invasively collected diagnostic or prognostic biomarker in either OPML or OSCC tissues – a significant finding given the urgent need for simpler and cheaper tests for oral cancer that circumvent the drawbacks of scalpel biopsy[8], [9].

Our results also suggest a mechanistic role for SLPI in oral cancer progression. Recent findings using *in-vitro* cell culture have suggested that SLPI inhibits the invasiveness of oral cancer cells[26]. Extending these findings, our results suggest a systemic decrease in SLPI, at least in oral epithelial cells, in patients susceptible to oral cancer development. This assertion was supported by about a 5-fold decrease in SLPI in matched healthy tissue compared to healthy normal controls, with a concordant decrease in its abundance in exfoliated cells in whole saliva. Determining the basis (e.g. genetic, epigenetic etc.) for this overall loss in SLPI abundance in patients developing OSCC will take more investigation.

Our results demonstrating that SLPI inhibits NF-κB transcriptional activity in-vitro in OPML cells further supports its mechanistic role in oral cancer progression. Increased NF-κB transcriptional activity, activating expression of pro-inflammatory cytokines, is a well-known factor in OSCC development[29], [30], [31]. A decrease in SLPI abundance may, at least in part, be a contributing factor to pro-inflammatory state leading to OSCC. Others have shown a role for SLPI as an inhibitor of NF-κB activity, demonstrating that it may disrupt the signaling pathway leading the NF-κB activation[36] and/or possibly compete for binding to DNA at NF-κB regulatory sites[32]. Our study is the first to show SLPI as an inhibitor of NF-κB in the context of oral cancer, specifically in a model OPML cell line. Understanding the exact mechanism of inhibition will take more investigation. Our observation opens the intriguing possibility of SLPI as a treatment option for at-risk OPML lesions, as such treatments are urgently needed to prevent development of invasive OSCC. SLPI offers possible advantages in such an application, as it is a small protein (approximately 11 kDa), known to be free of post-translational modifications, and shown to be taken up by cells readily[32].

Our findings generate a number of intriguing hypotheses for testing in the future. For one, the possibility of SLPI as a non-invasively collected diagnostic or prognostic biomarker could be tested in larger cohorts of OPML and OSCC patients. Its role as an inhibitor of NF-κB in OPML, with potential for preventive treatment could be tested and investigated in a number ways. Experiments examining the nature of inhibition, such as direct binding at NF-κB sites, and effects on gene and protein expression would be illuminating. Testing truncated versions of SLPI may also show increased inhibitory effects on NF-κB due to better cellular uptake and/or DNA binding. On a final note of interest, recent evidence also suggests a role for SLPI in inhibiting human papilloma virus (HPV) infection and subsequent head and neck cancer development[37]. Investigation on effects of SLPI treatment to HPV+ cancer cells would be of keen interest. The findings we present here provide a starting point for such future investigations, which could solidify SLPI as a highly valuable protein in the diagnosis, prognosis and treatment of oral cancer.

The mass spectrometry proteomics data have been deposited to the ProteomeXchange Consortium (http://proteomecentral.proteomexchange.org) via the PRIDE partner repository [38] with the dataset identifier PXD000807 and DOI 10.6019/PXD000807.

## Supporting Information

Figure S1 Coommassie stained images of total protein load (A) brushed oral cellsfrom patients; (B) exfoliated cellsfrom whole salivafrom patients; (C) Primary cells lines, including RhEK keratinocytes, MSK oral leukoplakia cells, and CA-9-22 oral cancer cells. These membranes were used for western blotting results shown in Figure 3 of text.
(DOC)

Table S1 Patient characteristics.
(DOC)

Table S2 All proteins identified and quantified in proteomic analyses. Results from the two separate iTRAQ reagent-bases proteomics analyses are shown in separate worksheets.
(XLSX)

## References

[1] Howlader N, Noone AM, Krapcho M, Garshell J, Neyman N, et al.. (2012) SEER Cancer Statistics Review, 1975–2010. National Cancer Institute.

[2] Rhodus NL (2005) Oral cancer: leukoplakia and squamous cell carcinoma. Dent Clin North Am 49: 143–165, ix. 10.1016/j.cden.2004.07.003 15567366

[3] Silverman S Jr (2001) Demographics and occurrence of oral and pharyngeal cancers. The outcomes, the trends, the challenge. J Am Dent Assoc 132 Suppl: 7S–11S. 11803655 10.14219/jada.archive.2001.0382

[4] Rhodus NL (2009) Oral cancer and precancer: improving outcomes. Compend Contin Educ Dent 30: 486–488, 490–484, 496–488 passim; quiz 504, 520. 19824564

[5] Lingen MW, Kalmar JR, Karrison T, Speight PM (2008) Critical evaluation of diagnostic aids for the detection of oral cancer. Oral Oncol 44: 10–22. 17825602 10.1016/j.oraloncology.2007.06.011 PMC2424250

[6] Axell T, Pindborg JJ, Smith CJ, van der Waal I (1996) Oral white lesions with special reference to precancerous and tobacco- related lesions: conclusions of an international symposium held in Uppsala, Sweden, May 18–21 1994. International Collaborative Group on Oral White Lesions. J Oral Pathol Med 25: 49–54. 8667255 10.1111/j.1600-0714.1996.tb00191.x

[7] Lumerman H, Freedman P, Kerpel S (1995) Oral epithelial dysplasia and the development of invasive squamous cell carcinoma. Oral Surg Oral Med Oral Pathol Oral Radiol Endod 79: 321–329. 7621010 10.1016/s1079-2104(05)80226-4

[8] Holmstrup P, Vedtofte P, Reibel J, Stoltze K (2007) Oral premalignant lesions: is a biopsy reliable? J Oral Pathol Med 36: 262–266. 17448135 10.1111/j.1600-0714.2007.00513.x

[9] Pentenero M, Carrozzo M, Pagano M, Galliano D, Broccoletti R, et al (2003) Oral mucosal dysplastic lesions and early squamous cell carcinomas: underdiagnosis from incisional biopsy. Oral Dis 9: 68–72. 12657031 10.1034/j.1601-0825.2003.02875.x

[10] Mercadante V, Paderni C, Campisi G (2012) Novel non-invasive adjunctive techniques for early oral cancer diagnosis and oral lesions examination. Curr Pharm Des 18: 5442–5451. 22632399 10.2174/138161212803307626

[11] Acha A, Ruesga MT, Rodriguez MJ, Martinez de Pancorbo MA, Aguirre JM (2005) Applications of the oral scraped (exfoliative) cytology in oral cancer and precancer. Med Oral Patol Oral Cir Bucal 10: 95–102. 15735540

[12] Mehrotra R, Gupta A, Singh M, Ibrahim R (2006) Application of cytology and molecular biology in diagnosing premalignant or malignant oral lesions. Mol Cancer 5: 11. 16556320 10.1186/1476-4598-5-11 PMC1448188

[13] Driemel O (2007) Protein profiling of oral brush biopsies: S100A8 and S100A9 can differentiate between normal, premalignant, and tumor cells. Proteomics Clin Appl 1: 486–493. 21136700 10.1002/prca.200600669

[14] Remmerbach TW, Maurer K, Janke S, Schellenberger W, Eschrich K, et al (2011) Oral brush biopsy analysis by matrix assisted laser desorption/ionisation-time of flight mass spectrometry profiling–a pilot study. Oral Oncol 47: 278–281. 21354855 10.1016/j.oraloncology.2011.02.005

[15] Zhang Y, Fonslow BR, Shan B, Baek MC, Yates JR 3rd (2013) Protein analysis by shotgun/bottom-up proteomics. Chem Rev 113: 2343–2394. 23438204 10.1021/cr3003533 PMC3751594

[16] Sacks PG (1996) Cell, tissue and organ culture as in vitro models to study the biology of squamous cell carcinomas of the head and neck. Cancer Metastasis Rev 15: 27–51. 8842478 10.1007/BF00049486

[17] Rhim JS (1989) Neoplastic transformation of human epithelial cells in vitro. Anticancer Res 9: 1345–1365. 2686534

[18] Wu JZ, Adachi I, Watanabe T (1991) Cytotoxic activity of FK973 against human oral and breast cancer cells. Chin Med J (Engl) 104: 834–837. 1661225

[19] Wisniewski JR, Zougman A, Nagaraj N, Mann M (2009) Universal sample preparation method for proteome analysis. Nat Methods 6: 359–362. 19377485 10.1038/nmeth.1322

[20] Yang F, Shen Y, Camp DG 2nd, Smith RD (2012) High-pH reversed-phase chromatography with fraction concatenation for 2D proteomic analysis. Expert Rev Proteomics 9: 129–134. 22462785 10.1586/epr.12.15 PMC3337716

[21] Olsen JV, Schwartz JC, Griep-Raming J, Nielsen ML, Damoc E, et al (2009) A dual pressure linear ion trap Orbitrap instrument with very high sequencing speed. Mol Cell Proteomics 8: 2759–2769. 19828875 10.1074/mcp.M900375-MCP200 PMC2816009

[22] Onsongo G, Stone MD, Van Riper SK, Chilton J, Wu B, et al (2010) LTQ-iQuant: A freely available software pipeline for automated and accurate protein quantification of isobaric tagged peptide data from LTQ instruments. Proteomics 10: 3533–3538. 20821806 10.1002/pmic.201000189 PMC3025484

[23] Ross PL, Huang YN, Marchese JN, Williamson B, Parker K, et al (2004) Multiplexed protein quantitation in Saccharomyces cerevisiae using amine-reactive isobaric tagging reagents. Mol Cell Proteomics 3: 1154–1169. 15385600 10.1074/mcp.M400129-MCP200

[24] Ting L, Rad R, Gygi SP, Haas W (2011) MS3 eliminates ratio distortion in isobaric multiplexed quantitative proteomics. Nat Methods 8: 937–940. 21963607 10.1038/nmeth.1714 PMC3205343

[25] Wenger CD, Lee MV, Hebert AS, McAlister GC, Phanstiel DH, et al (2011) Gas-phase purification enables accurate, multiplexed proteome quantification with isobaric tagging. Nat Methods 8: 933–935. 21963608 10.1038/nmeth.1716 PMC3205195

[26] Wen J, Nikitakis NG, Chaisuparat R, Greenwell-Wild T, Gliozzi M, et al (2011) Secretory leukocyte protease inhibitor (SLPI) expression and tumor invasion in oral squamous cell carcinoma. Am J Pathol 178: 2866–2878. 21641406 10.1016/j.ajpath.2011.02.017 PMC3124294

[27] Gumus ZH, Du B, Kacker A, Boyle JO, Bocker JM, et al (2008) Effects of tobacco smoke on gene expression and cellular pathways in a cellular model of oral leukoplakia. Cancer Prev Res (Phila) 1: 100–111. 19138943 10.1158/1940-6207.CAPR-08-0007 PMC3773527

[28] Gamou S, Shimizu N (1987) Change in metabolic turnover is an alternate mechanism increasing cell surface epidermal growth factor receptor levels in tumor cells. J Biol Chem 262: 6708–6713. 3571280

[29] Duffey DC, Chen Z, Dong G, Ondrey FG, Wolf JS, et al (1999) Expression of a dominant-negative mutant inhibitor-kappaBalpha of nuclear factor-kappaB in human head and neck squamous cell carcinoma inhibits survival, proinflammatory cytokine expression, and tumor growth in vivo. Cancer Res 59: 3468–3474. 10416612

[30] Molinolo AA, Amornphimoltham P, Squarize CH, Castilho RM, Patel V, et al (2009) Dysregulated molecular networks in head and neck carcinogenesis. Oral Oncol 45: 324–334. 18805044 10.1016/j.oraloncology.2008.07.011 PMC2743485

[31] Patel A, Miller L, Ahmed K, Ondrey F (2004) NF-Kappa-B downregulation strategies in head and neck cancer treatment. Otolaryngol Head Neck Surg 131: 288–295. 15365549 10.1016/j.otohns.2004.03.004

[32] Taggart CC, Cryan SA, Weldon S, Gibbons A, Greene CM, et al (2005) Secretory leucoprotease inhibitor binds to NF-kappaB binding sites in monocytes and inhibits p65 binding. J Exp Med 202: 1659–1668. 16352738 10.1084/jem.20050768 PMC2212970

[33] Wang N, Thuraisingam T, Fallavollita L, Ding A, Radzioch D, et al (2006) The secretory leukocyte protease inhibitor is a type 1 insulin-like growth factor receptor-regulated protein that protects against liver metastasis by attenuating the host proinflammatory response. Cancer Res 66: 3062–3070. 16540655 10.1158/0008-5472.CAN-05-2638

[34] Caicedo-Granados EE, Wuertz BR, Marker PH, Lee GS, Ondrey FG (2011) The effect of indomethacin on paclitaxel sensitivity and apoptosis in oral squamous carcinoma cells: the role of nuclear factor-kappaB inhibition. Arch Otolaryngol Head Neck Surg 137: 799–805. 21844414 10.1001/archoto.2011.131

[35] Scott A, Weldon S, Taggart CC (2011) SLPI and elafin: multifunctional antiproteases of the WFDC family. Biochem Soc Trans 39: 1437–1440. 21936829 10.1042/BST0391437

[36] Taggart CC, Greene CM, McElvaney NG, O’Neill S (2002) Secretory leucoprotease inhibitor prevents lipopolysaccharide-induced IkappaBalpha degradation without affecting phosphorylation or ubiquitination. J Biol Chem 277: 33648–33653. 12084717 10.1074/jbc.M203710200

[37] Quabius ES, Moller P, Haag J, Pfannenschmidt S, Hedderich J, et al.. (2013) The role of the antileukoprotease SLPI in smoking-induced human papillomavirus-independent head and neck squamous cell carcinomas. Int J Cancer. 10.1002/ijc.28462 23996702

[38] Vizcaino JA, Cote RG, Csordas A, Dianes JA, Fabregat A, et al (2013) The PRoteomics IDEntifications (PRIDE) database and associated tools: status in 2013. Nucleic Acids Res 41: D1063–1069. 23203882 10.1093/nar/gks1262 PMC3531176

