# Supplementary material for: Quantitative Proteomic Analysis of Oral Brush Biopsies Identifies Secretory Leukocyte Protease Inhibitor as a Promising, Mechanism-Based Oral Cancer Biomarker
Source: PLoS One. 2014 Apr 18;9(4):e95389. doi: 10.1371/journal.pone.0095389 (PMC3991667; doi:10.1371/journal.pone.0095389)

**Supplemental Figure 1.** Coommassie stained images of total protein load (A) brushed oral cells from patients; (B) exfoliated cells from whole saliva from patients; (C) Primary cells lines, including RhEK keratinocytes, MSK oral leukoplakia cells, and CA-9-22 oral cancer cells. These membranes were used for western blotting results shown in Figure 3 of text.


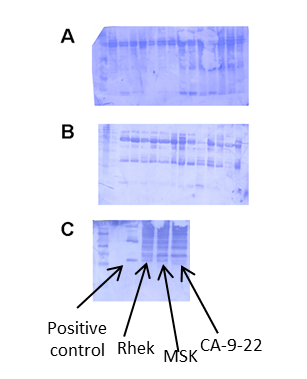

Supplement: Figure S1 — Coommassie stained images of total protein load (A) brushed oral cellsfrom patients; (B) exfoliated cellsfrom whole salivafrom patients; (C) Primary cells lines, including RhEK keratinocytes, MSK oral leukoplakia cells, and CA-9-22 oral cancer cells. These membranes were used for western blotting results shown in Figure 3 of text. (DOC) [file pone.0095389.s001.doc]
