# Supplementary material for: Quantitative Proteomic Analysis of Oral Brush Biopsies Identifies Secretory Leukocyte Protease Inhibitor as a Promising, Mechanism-Based Oral Cancer Biomarker
Source: PLoS One. 2014 Apr 18;9(4):e95389. doi: 10.1371/journal.pone.0095389 (PMC3991667; doi:10.1371/journal.pone.0095389)
Supplement: Table S1 — Patient characteristics. (DOC) [file pone.0095389.s002.doc]

**Supplemental Table 1.** Patient characteristics

|  |  |  |  |  | Tobacco | TNM |
| --- | --- | --- | --- | --- | --- | --- |
| ID | Lesion site | Clinical diagnosis | age | Sex | exposure | stage |
| 1 | Under anterior part of tongue | leukoplakia | 79 | M | Former Smoker | n/a |
| 2 | Right tongue | leukoplakia | 61 | F | Never Smoked | n/a |
| 3 | Left ventro-lateral tongue | high grade squamous dysplasia, carcinoma in situ | 60 | M | Never Smoked | n/a |
| 4 | Left ventral lateral tongue | leukoplakia | 53 | M | Former Smoker | n/a |
| 5 | Gingival lingual mucosa near lower incisor | Leuloplakia | 50 | F | Current Smoker | n/a |
| 6 | Left lateral tongue | Leuloplakia | 60 | F | Never Smoked | n/a |
| 7 | 2cm posterior to tip of tongue, directly on midline | benign squamous mucosa | 48 | M | Former Smoker | n/a |
| 8 | Anterior floor of mouth | high grade squamous dysplasia (pre-neoplastic carcinoma in situ) | 60 | M | Current Smoker | n/a |
| 9 | Tongue | Leuloplakia | 68 | F | Former Smoke | n/a |
| 10 | Tongue | Leuloplakia | 61 | F | Current Smoker | n/a |
| 11 | Cheek Mucosa | Leuloplakia | 54 | M | Former Smoker | n/a |
| 12 | Right floor of mouth involving mandible | SCC | 66 | M | Former Smoker | T4N0M0 |
| 13 | left mobile tongue | SCC | 42 | F | Never Smoked | T2 |
| 14 | Right soft palate retromolar trigone | SCC | 71 | F | Former Smoker | T2 |
| 15 | Base of tongue | SCC | 51 | F | Current Smoker | T2N2cM0 |
| 16 | Right tongue | SCC | 68 | M | Former Smoker | T2 |
| 17 | Right retromolar trigone | SCC | 60 | M | Current Smoker | T4N2bMx |
| 18 | Right Mandibular Mucosa | SCC | 43 | M | Current Smoker | T4N2bM0 |
| 19 | Right buccal mucosa involving right maxilla | SCC | 45 | M | Former Smoker | T4a/bN1M0 |
| 20 | Left lateral tongue and floor of mouth | SCC | 69 | M | Former Smoker | T4N0M0 |
| 21 | right mandibular alveolus | SCC | 88 | F | Never Smoked | T4N 2b |
| 22 | Right retromolar trigone | SCC | 62 | F | Former Smoker | T2N0M0 |
| 23 | Floor of mouth | Control 01 | 62 | M | Former Smoker | n/a |
| 24 | Retromolar Trigone | Control 02 | 63 | F | Former Smoker | n/a |
| 25 | Retromolar Trigone | Control 03 | 70 | F | Former Smoker | n/a |
| 26 | Mandibular Mucosa Tongue | Control 04 | 40 | M | Never Smoker; CurrentChewing tobacco user | n/a |
| 27 | Right tongue | Control 05 | 40 | M | Never Smoker | n/a |
| 28 | Right mandibular | Control 06 | 66 | F | Never Smoker | n/a |
| 29 | Alveolus Right cheek mucosa | Control 07 | 84 | F | Never Smoker | n/a |
| 30 | Right cheek mucosa | Control 08 | 62 | M | Never Smoker | n/a |
| 31 | Right cheek mucosa | Control 09 | 58 | F | Never Smoker | n/a |
| 32 | Left cheek mucosa | Control 10 | 64 | M | Never Smoker | n/a |
